# The Association of Free Testosterone with Sarcopenic Obesity in Community-Dwelling Older Men: A Cross-Sectional Study

**DOI:** 10.3390/medicina60050754

**Published:** 2024-05-01

**Authors:** Seongmin Choi, Jinmann Chon, Myung Chul Yoo, Ga Yang Shim, Miji Kim, Yunsoo Soh, Chang Won Won

**Affiliations:** 1 Department of Physical Medicine and Rehabilitation Medicine, Kyung Hee University Medical Center, Seoul 02477, Republic of Korea; ysisminee@naver.com (S.C.); kkangmann@naver.com (J.C.); famousir@naver.com (M.C.Y.); wholhear@gmail.com (G.Y.S.); 2 Department of Physical Medicine and Rehabilitation, Graduate School, Kyung Hee University, Seoul 02447, Republic of Korea; 3 Department of Biomedical Science and Technology, College of Medicine, East-West Medical Research Institute, Kyung Hee University, Seoul 02447, Republic of Korea; mijiak@khu.ac.kr; 4 Department of Family Medicine, Kyung Hee University Medical Center, Seoul 02447, Republic of Korea

**Keywords:** testosterone, sarcopenic obesity, aging

## Abstract

*Background and Objectives*: Sarcopenic obesity, a clinical condition coexisting with obesity and sarcopenia, is associated with a high risk of functional impairment, reduced quality of life, and increased mortality. A decline in age-related free testosterone (FT) levels has been reported to be associated with decreased muscle mass and muscle strength and increased fat mass. However, the association between low FT levels and risk of sarcopenic obesity has not been well studied. This study aimed to investigate the direct association between low FT levels and sarcopenic obesity. *Materials and Methods*: This cross-sectional study used data of 982 community-dwelling men aged 70–84 years from the Korean Frailty and Aging Cohort Study. Sarcopenia was defined according to the criteria of the Asian Group for Sarcopenia (AWGS) 2019. Obesity was defined as a body fat mass ≥28.3%. Participants who met both sarcopenia and obesity criteria were defined as having sarcopenic obesity. Low FT levels were defined as FT levels <17.35 pmol/L according to the Endocrine Society Clinical Practice Guidelines. *Results*: The prevalence of sarcopenia, obesity, and sarcopenic obesity was significantly higher in the low-FT group than in the normal-FT group. Low FT levels were significantly associated with a higher risk of obesity (odds ratio [OR], 2.09, 95% confidence interval [CI], 1.11–3.92), sarcopenia (2.57, 95% CI 1.08–6.10), and sarcopenic obesity (3.66, 95% CI 1.58–8.47) compared with the healthy control group. The risk of low appendicular skeletal muscle mass index (ASMI) (1.78, 95% CI 1.04–3.02) and high fat mass (1.92, 95% CI 1.12–3.31) was significantly higher in the low-FT group than in the normal-FT group. *Conclusions*: This study showed that low FT levels were associated with a higher risk of sarcopenic obesity. Low FT levels were mainly related to body composition parameters such as low ASMI and high fat mass.

## 1. Introduction

Sarcopenic obesity is a clinical condition that coexists with obesity and sarcopenia [1]. Aging is accompanied by a loss of skeletal muscle mass and physical function and an increase in body fat, leading to the development of sarcopenic obesity [2]. Sarcopenic obesity has a higher risk of functional impairment, reduced quality of life, metabolic disease comorbidities, and increased mortality than sarcopenia or obesity alone [3,4]. Therefore, it is important to identify the risk factors of sarcopenic obesity to aid in its prevention and treatment. The etiology of sarcopenic obesity is multifactorial and includes sedentary behavior, unhealthy diet, chronic low-grade inflammation, and sex-specific hormonal changes [4].

Testosterone, a sex hormone, is produced by Leydig cells in response to luteinizing hormones [5]. Testosterone binds to sex hormone-binding globulin or albumin, and only 1–2% of testosterone exists in its free form. Free testosterone (FT), the active form of testosterone taken up by target cells, binds to the androgen receptor (AR). Several ARs expressed in muscle play important roles in maintaining muscle strength and muscle mass [6,7]. Serum testosterone levels decrease by 2–3% annually with age in men [8]. A decline in age-related FT levels is associated with decreased muscle mass and muscle strength and increased fat mass [9,10,11,12]. Baumgartner et al. found a positive correlation between serum FT levels and appendicular muscle mass. [9] A cross-sectional study by Kong et al., which included 922 men, reported weak muscle strength in the lower-FT-level group [10]. Regarding the association between FT levels and obesity, Travison et al. revealed that high body mass index (BMI) accelerated serum testosterone decline [11]. Furthermore, a longitudinal study of community-dwelling men showed that the loss of body weight was associated with increased testosterone levels [12].

However, the effect of low FT levels on the risk of developing sarcopenic obesity has not been thoroughly studied. To our knowledge, only one study has investigated the direct effect of low FT levels on sarcopenic obesity and reported the absence of an association between these two factors [13]. However, this earlier study was limited by an exceedingly small sample size and the fact that salivary testosterone levels are not directly comparable to serum testosterone levels because salivary testosterone binds to salivary proteins [14].

Considering that low FT levels are independently associated with obesity and sarcopenia, we hypothesized that low FT levels might be directly associated with sarcopenic obesity. Identifying the association between FT levels and sarcopenic obesity might be helpful to prevent and treat sarcopenic obesity. In this large cross-sectional study of healthy community-dwelling older adults, we aimed to investigate the direct association between low FT levels and sarcopenic obesity. We used the data from the Korean Frailty and Aging Cohort Study (KFACS) in this study.

## 2. Materials and Methods

### 2.1. Study Population

**Figure 1:**
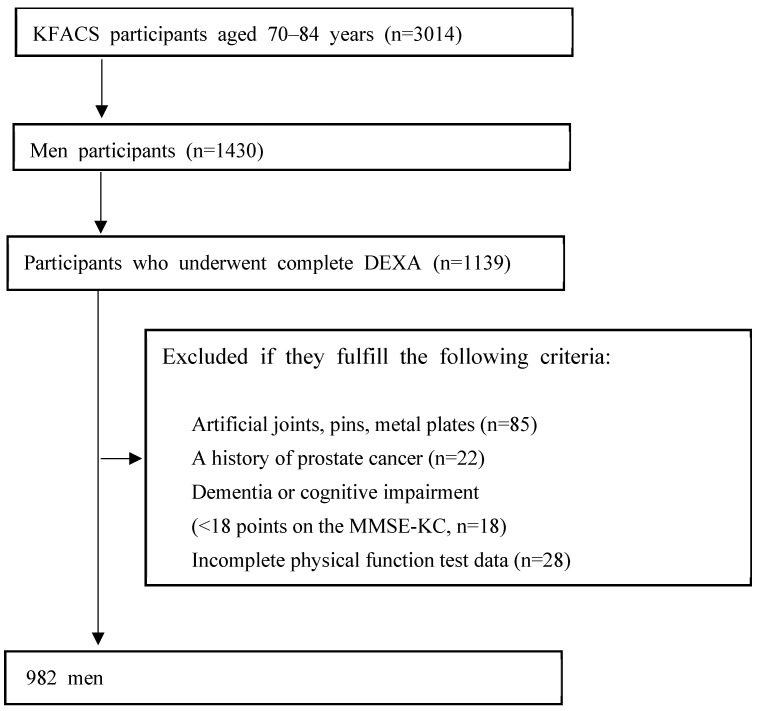
Flow chart of the participant recruitment process. DEXA, dual-energy X-ray absorptiometry; KFACS, Korean Frailty and Aging Cohort Study; MMSE-KC, Mini-Mental State Examination of the Korean version of the Consortium to Establish a Registry for Alzheimer’s Disease (CERAD) Assessment Packet.

This cross-sectional study used data from the KFACS collected between 2016 and 2017. The KFACS is a longitudinal nationwide cohort study conducted at 10 centers, comprising 8 medical centers and 2 public health centers. A total of 3014 community-dwelling older adults aged 70–84 were recruited. At each clinical site of the 10 study centers, a survey including questionnaires, health examinations, face-to-face interviews, and laboratory tests was conducted. The participants included 1430 men, and 1139 of them had undergone dual-energy X-ray absorptiometry (DXA). The exclusion criteria were as follows: participants with artificial joints or other metal objects in the appendicular body regions (n = 85); a history of or current prostate cancer (n = 22), dementia, or cognitive impairment (<18 points on the Mini-Mental Status Examination in the Korean version [MMSE-KC] of the Consortium to Establish a Registry for Alzheimer’s Disease [CERAD] assessment packet) (n = 18); incomplete physical function test (n = 28); or a history of stroke or hemiplegia (n = 4). A total of 982 older men were included in this study (Figure 1). The KFACS protocol was approved by the Institutional Review Board (IRB) of the Clinical Research Ethics Committee of the Kyung Hee University Medical Center (IRB approval number: 2015-12-103).

### 2.2. Definitions

#### 2.2.1. Sarcopenia

Sarcopenia was evaluated according to the Asian Working Group for Sarcopenia (AWGS) 2019 diagnostic criteria [15]. Participants with low skeletal muscle mass and either low muscle strength or physical performance were defined as having sarcopenia.

(1) Skeletal muscle mass: we used DXA to measure appendicular skeletal muscle mass (ASM), and the ASM index (ASM/height^2^, ASMI) was calculated to compare muscle mass according to height (cutoff value, men: <7.0 kg/m^2^) [15].
(2) Muscle strength: Handgrip strength (HGS) was measured using a hand dynamometer (T.K.K.5401, Takei Scientific Instruments Co., Ltd., Tokyo, Japan). HGS measurements were performed twice on both sides with the elbow extended in a standing position. The participants were instructed to hold the grip for 3 s with full force, and the maximum value was obtained in kilograms (cutoff value: <28 kg) [15].
(3) Physical performance: A short physical performance battery (SPPB) was used to evaluate the physical performance. The SPPB is a widely used test that comprehensively assesses physical performance in the elderly population. The test consisted of three standing balance measures, a 4 m gait speed measure, and five sit-to-stand tests. Each item was scored from 0 to 4 points based on the established population for epidemiological studies of the elderly, with a maximum score of 12 points [16]. A score of ≤9 points was defined as low physical performance according to the AWGS diagnostic criteria [15].

#### 2.2.2. Obesity

While there is no standardized definition of obesity based on body fat percentage, the commonly used definition of obesity for older adults is the criterion suggested by a previous New Mexico aging process study. Obesity was defined as a percentage of body fat ≥ the 60th percentile in the study population, and this corresponds to a cutoff value of ≥28% in men [17]. Obesity was defined as a percentage of body fat ≥ the 60th percentile of our study population according to the criteria of the New Mexico aging process study, and this had a cutoff value of ≥28.3%, similar to that of the New Mexico aging process study.

The participants were divided into four groups based on their sarcopenia and obesity status: healthy controls (non-sarcopenic and non-obese), sarcopenia (sarcopenic and non-obese), obesity (non-sarcopenic and obese), and sarcopenic obesity (sarcopenic and obese).

### 2.3. Measurement of Free Testosterone

Blood samples were collected between 0730 and 0800 h after overnight fasting. Serum FT levels were measured using a radioimmunoassay (AMP 10-R4100). The inter-assay coefficients of variation (CVs) were 9.3%, 5.7%, and 11.7%, and the intra-assay CVs were 19.5%, 7.3%, and 9.1% for the low, medium, and high pools, respectively. The FT concentrations were measured in conventional units (pg/mL), with a detection limit of 0.40 pg/mL, and were multiplied by 3.47 to convert to SI units (pmol/L). Low FT levels were defined as <17.35 pmol/L according to the Endocrine Society Clinical Practice Guidelines [18].

### 2.4. Other Measurements

Trained investigators obtained sociodemographic data and medical histories, including age, location of residence, sex, height, weight, BMI, waist circumference, comorbidities, and smoking and alcohol consumption status. Participants who smoked more than one cigarette per week were defined as smokers and those who drank alcohol more than once per week were defined as alcohol consumers. The amount of physical activity was assessed by the short-form International Physical Activity Questionnaire (IPAQ) [19]. The IPAQ consists of questions regarding the duration and frequency of weekly physical activity. The total physical activity level was calculated by multiplying the time (min) and frequency of physical activity (times per week) and expressed as metabolic equivalents (METs). Participants were classified into three groups based on physical activity: high activity (≥3000 MET-min/week), moderate activity (600–2999 MET-min/week), and low activity (<600 MET-min/week) [19]. Nutritional status was assessed using the Mini-Nutritional Assessment Short Form (MNA-SF). The MNA-SF is a validated test that has been used for short-term malnutrition screening [20].

### 2.5. Confounding Variables

Potential confounding variables include age, hypertension, dyslipidemia, heart disease, osteoarthritis, osteoporosis, chronic obstructive pulmonary disease (COPD), cerebrovascular accident, diabetes mellitus, alcohol consumption history, smoking history, heart disease, MNA-SF, physical activity, and MMSE-KC score. In our study, the selection of confounding variables was based on previous studies of the relationship between exposure and outcomes [21,22,23,24].

### 2.6. Statistical Analysis

Continuous and categorical variables were compared using the *t*-test, Mann–Whitney U test, and Pearson’s chi-square test. Unadjusted and fully adjusted logistic regression analyses were performed, and odds ratios (ORs) and 95% confidence intervals (CIs) were calculated. The fully revised analysis was adjusted for potential confounding variables including age, hypertension, dyslipidemia, heart disease, osteoarthritis, osteoporosis, chronic obstructive pulmonary disease (COPD), cerebrovascular accident, diabetes mellitus, alcohol consumption history, smoking history, heart disease, MNA-SF, physical activity, and MMSE-KC score to address confounding bias. Statistical analyses were performed using the Statistical Package for Social Sciences (version 25.0; SPSS Inc., Chicago, IL, USA), and *p* < 0.05 was considered statistically significant.

## 3. Results

The baseline characteristics of the participants with respect to FT levels are presented in Table 1. Among the 982 participants, 910 (92.7%) were in the normal-FT group and 72 (7.3%) were in the low-FT group. The HGS was significantly lower in the low-FT group than in the normal-FT group (normal-FT group, 32.64 ± 5.68; low-FT group, 30.79 ± 5.41, *p* < 0.01). ASMI was significantly lower (normal-FT group, 7.07 ± 0.84; low-FT group, 6.80 ± 0.88, *p* < 0.01) and fat mass was significantly higher in the low-FT group than in the normal-FT group (normal-FT group, 0.26 ± 0.06; low-FT group, 0.29 ± 0.06). Other characteristics, such as age, waist circumference, fasting glucose level, and glycated hemoglobin A1c (HbA1c) levels, were significantly higher in the low-FT group than in the normal-FT group.

**Figure 2:**
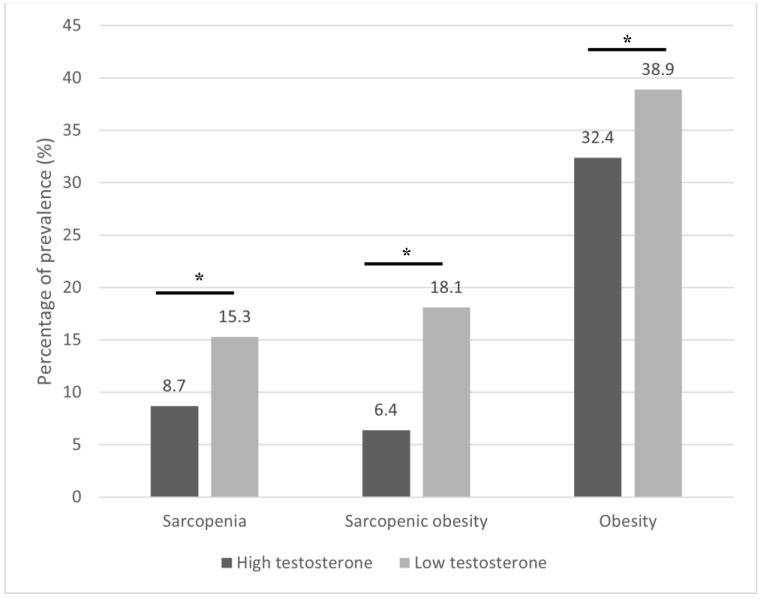
Percentage of prevalence of sarcopenia, obesity, and sarcopenic obesity with respect to free testosterone levels. * *p* < 0.01.

Figure 2 shows the prevalence of diseases with respect to the FT levels. The prevalence of sarcopenia (normal-FT group, 8.7%; low-FT group, 15.3%; *p* < 0.01), sarcopenic obesity (normal-FT group, 6.4%; low-FT group, 18.1%; *p* < 0.01), and obesity (normal-FT group, 32.4%; low-FT group, 38.9%; *p* < 0.01) was significantly higher in the low-FT group than in the normal-FT group.

**Figure 3:**
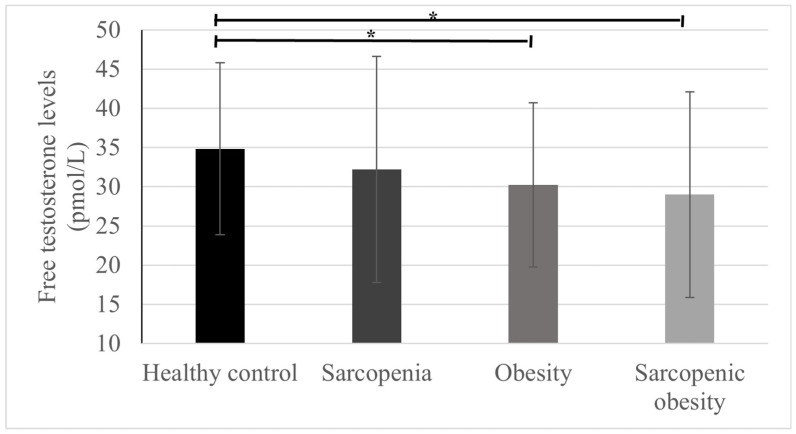
Mean and standard deviation of free testosterone levels according to group. * *p* < 0.01.

Figure 3 shows the FT levels according to group. FT levels were significantly lower in the obesity group (healthy control, 34.82 ± 10.96; obesity, 30.24 ± 10.48, *p* < 0.01) and sarcopenic obesity group (healthy control 34.82 ± 10.96; sarcopenic obesity, 29.00 ± 13.11, *p* < 0.01) than in the healthy control group. In the sarcopenia group, FT levels were not significantly lower than those in the healthy control group (healthy control 34.82 ± 10.96; sarcopenic obesity, 32.19 ± 14.42, *p* = 0.10).

The results of the multivariate logistic regression analyses of the association among circulating FT levels and sarcopenia, obesity, and sarcopenic obesity are shown in Table 2. After adjusting for confounding variables, low FT levels were significantly associated with a higher risk of obesity (odds ratio [OR], 2.09; 95% confidence interval [CI], 1.11–3.92), sarcopenia (OR, 2.57; 95% CI, 1.08–6.10), and sarcopenic obesity (OR, 3.66; 95% CI, 1.58–8.47) compared with the healthy control group.

Table 3 shows the logistic regression analysis of the association between FT levels and the individual components of sarcopenic obesity. In this multivariate analysis, the risk of low ASMI (OR, 1.78; 95% CI, 1.04–3.02) and high fat mass (OR, 1.92; 95% CI, 1.12–3.31) was significantly higher in the low-FT group than in the normal-FT group.

## 4. Discussion

This study investigated the association between low FT levels, sarcopenic obesity, and individual components of sarcopenic obesity. We found that low FT levels were associated with a higher risk of obesity, sarcopenia, and especially sarcopenic obesity. Regarding the individual components of sarcopenic obesity, low FT levels were correlated with body composition, such as lower ASMI and higher fat mass, rather than with muscle strength and physical performance.

Although a few studies have failed to show a positive effect of testosterone on muscle strength [25,26], the current evidence has established a negative effect of low testosterone levels on muscle mass and strength [6]. In one cross-sectional study, serum FT levels were significantly correlated with appendicular muscle mass [9]. A study of 3875 men in China reported a positive correlation between appendicular lean mass and testosterone levels [27]. In a study of 403 men in the Netherlands, grip strength and leg extensor strength were significantly correlated with testosterone levels [10]. Further, in our previous cross-sectional study, low serum FT levels were associated with the prevalence of sarcopenia in men as defined by the AWGS 2019 criteria [7].

An association between circulating testosterone levels and obesity has been reported previously. Previous studies have consistently demonstrated a strong association between obesity and low testosterone levels. A prospective Massachusetts Male Aging Study of 1667 men showed that increased BMI accelerated the decline in serum testosterone levels [11]. Similar results have been reported in other cohort studies in Australia and Europe [28,29]. Furthermore, in a longitudinal study of 2,736 community-dwelling men, weight loss was associated with increased testosterone levels [12]. While these studies found that obesity reduces testosterone levels, other reports have revealed that low testosterone levels promote obesity. A longitudinal study of 110 Japanese American men found that lower baseline testosterone levels predicted an increase in abdominal fat after 7.5 years of follow-up [30]. In addition, multiple randomized controlled trials have verified that testosterone treatment reduces fat mass [31,32,33].

Although an independent association between low testosterone levels and either sarcopenia or obesity has been confirmed in previous studies, only one study has investigated the direct association between testosterone levels and sarcopenic obesity. A cross-sectional study of 190 community-dwelling older adults examined the relationship between salivary testosterone levels and the risk of sarcopenic obesity and failed to reveal a significant effect of salivary testosterone levels on sarcopenic obesity [13]. This study has a limitation: salivary testosterone levels are not directly comparable to serum testosterone levels because salivary testosterone is known to bind to salivary proteins [14].

In contrast to a previous report, we found that low FT levels were associated with a higher risk of sarcopenic obesity, and this association was stronger than that of either sarcopenia or obesity alone. The association between low FT levels and sarcopenic obesity was due to negative effects on body composition, such as low ASMI and high fat mass. The role of testosterone in regulating metabolism and its effects on body composition have been revealed in previous studies. The association between low testosterone levels and high fat mass has been consistently reported, and the relationship was found to be bidirectional [34]. Increased body fat mass suppresses the hypothalamic–pituitary–testicular axis via increased pro-inflammatory cytokines and insulin resistance, whereas low testosterone levels promote the further accumulation of fat mass through decreased lipolysis and β-oxidation and increased lipogenesis and adipocyte differentiation [35]. Low FT levels also cause muscle atrophy via both decreased anabolic and increased catabolic effects. Decreased anabolic effects are mediated by reduced myogenesis, differentiation of satellite cells, and protein synthesis, and increased catabolic effects are associated with enhanced muscle atrogin-1 and RING-finger protein-1 expression [6]. In addition, visceral fat deposition due to low FT levels contributes to an increase in inflammatory cytokines, such as interleukin-6 and tumor necrosis factor, which have catabolic effects on skeletal muscles [36]. As low FT levels affect both fat and muscle metabolism, sarcopenic obesity may be strongly associated with low FT levels.

The results of this nationwide, large cohort study of community-dwelling older adults are reasonable because reliable measurements of FT levels were used. This is the first study to investigate the association between low FT levels and sarcopenic obesity. However, this study has a few limitations. First, a causal relationship between low FT levels and sarcopenic obesity could not be determined due to the cross-sectional nature of the study design. Second, serum testosterone levels are affected by diurnal, pulsatile, and circannual rhythms [37]. Although we did not consider these biological rhythms of testosterone levels, we consistently measured testosterone levels early in the morning in accordance with the Endocrine Society CSlinical Practice Guidelines [18] and the need for clinicians to depend on conveniently obtained single samples. Finally, the results of this study may not be applicable to other races because it was conducted in a single race, namely, the East Asian Korean population. Since body composition differs among ethnicities, further prospective studies in other ethnicities are required to confirm our findings [38]

## 5. Conclusions

To our knowledge, this is the first large cross-sectional study to investigate the direct association between low FT levels and sarcopenic obesity. Our findings show that low FT levels are associated with a higher risk of sarcopenic obesity. This association is mainly attributed to the negative effects of low FT levels on body composition. It might be helpful to consider hypogonadism as one of the risk factors in patients with sarcopenic obesity in men.

## Figures and Tables

**Table 1:** Baseline characteristics of the participants with respect to free testosterone levels.

	Non-Low Free Testosterone (n = 910, 92.7%)	Low Free Testosterone † (n = 72, 7.3%)	*p*-Value
Age	76.16 ± 3.95	78.15 ± 3.41	<0.01 *
Height (cm)	164.99 ± 5.52	164.04 ± 6.16	0.16
Weight (kg)	65.27 ± 8.87	65.23 ± 9.02	0.97
BMI (kg/m^2^)	23.95 ± 2.84	24.22 ± 2.85	0.45
Waist circumference (cm)	88.52 ± 8.29	90.95 ± 9.01	0.02 *
Hypertension (n, %)	483 (53.1)	38 (52.8)	0.96
Diabetes mellitus (n, %)	218 (24)	23 (31.9)	0.13
Dyslipidemia (n, %)	219 (24.1)	23 (31.9)	0.14
Heart disease (n, %)	87 (9.6)	8 (11.1)	0.67
Osteoarthritis (n, %)	96 (10.5)	10 (13.9)	0.38
Osteoporosis (n, %)	25 (2.7)	3 (4.2)	0.49
CVA (n, %)	51 (5.6)	5 (6.9)	0.64
COPD (n, %)	18 (2.0)	0 (0.0)	0.23
Alcohol (n, %)	310 (34.1)	24 (33.3)	0.90
Current smoker (n, %)	99 (10.9)	7 (9.7)	0.76
Urban residence (n, %)	723 (79.5)	57 (79.2)	0.95
MMSE-KC	26.55 ± 2.44	26.43 ± 2.23	0.70
MNA-SF	12.93 ± 1.45	12.87 ± 1.55	0.76
Moderate-to-high physical activity (n, %)	834 (91.6)	63 (87.5)	0.23
Biochemical variables			
Free testosterone levels (pmol/L)	34.38 ± 10.04	10.82 ± 5.48	<0.01 *
Fasting glucose (mg/dL)	104.46 ± 23.50	114.36 ± 27.04	<0.01 *
HbA1c (%)	5.97 ± 0.81	6.17 ± 0.89	0.04 *
HOMA-IR	2.07 ± 4.86	2.75 ± 3.07	0.24
Total cholesterol (mg/dL)	168.42 ± 34.81	169.43 ± 38.78	0.81
HDL-C (mg/dL)	50.57 ± 14.23	52.38 ± 15.02	0.30
LDL-C (mg/dL)	104.55 ± 31.66	103.93 ± 35.16	0.16
TG (mg/dL)	116.57 ± 66.22	117.50 ± 47.17	0.91
Skeletal muscle function			
HGS, kg	32.64 ± 5.68	30.79 ± 5.41	<0.01 *
SPPB	11.19 ± 1.26	10.96 ± 1.28	0.13
Body composition			
ASMI	7.07 ± 0.84	6.80 ± 0.88	<0.01 *
Fat mass, %	26.31 ± 5.89	29.11 ± 6.08	<0.01 *

Abbreviations: ASMI, appendicular skeletal muscle mass index; BMI, body mass index; COPD, chronic obstructive pulmonary disease; CVA, cerebrovascular accident; HbA1c, glycated hemoglobin A1c percentage; HDL-C, high-density lipoprotein cholesterol; HGS, hand grip strength; HOMA-IR, homeostatic model assessment for insulin resistance; LDL-C, low-density lipoprotein cholesterol; MMSE-KC, Mini-Mental Status Examination-Korean version; MNA-SF, Mini Nutritional Assessment-Short Form; SPPB, short physical performance battery; TG, triglyceride. † Low free testosterone: <17.35 pmol/L. Categorical variables are reported as numbers (%) and were compared using Pearson’s chi-square test. Continuous variables are reported as means ± standard deviation. * *p* < 0.05.

**Table 2:** Logistic regression analysis of low free testosterone level (¶) predicting sarcopenia, obesity, and sarcopenic obesity.

	Unadjusted Model	Fully Adjusted Model
	OR (95% CI)	OR (95% CI)
Healthy control †	1.00 (reference)	1.00 (reference)
Obesity †	2.71 (1.12–3.94) *	2.09 (1.11–3.92) *
Sarcopenia †	3.32 (1.52–7.25) **	2.57 (1.08–6.10) *
Sarcopenic obesity †	5.35 (2.51–11.36) **	3.66 (1.58–8.47) **

Abbreviations: CI, confidence interval; OR, odds ratio. The fully adjusted model was adjusted for age, hypertension, dyslipidemia, heart disease, osteoarthritis, osteoporosis, chronic obstructive pulmonary disease (COPD), cerebrovascular accident, diabetes mellitus, alcohol history, smoking history, Mini Nutritional Assessment-Short Form (MNA-SF) score, physical activity, and Mini-Mental Status Examination-Korean version (MMSE-KC) score. ¶ Low free testosterone level: <17.35 pmol/L. † Healthy control, does not meet both the sarcopenia and obesity criteria; obesity, >28.3% of fat mass; sarcopenia, low appendicular skeletal muscle mass index (ASMI) (<7.0 kg/m^2^) and either a low hand grip strength (HGS) (<28 kg) or low physical performance (short physical performance battery [SPPB] score ≤ 9); sarcopenic obesity, meet both sarcopenia and obesity criteria. * *p* < 0.05, ** *p* < 0.01.

**Table 3:** Logistic regression analysis of low free testosterone levels predicting individual components of sarcopenic obesity.

	Unadjusted Model	Fully Adjusted Model
	OR (95% CI)	OR (95% CI)
Low HGS †	2.11 (1.27–3.52) **	1.50 (0.85–2.63)
Low SPPB †	1.92 (0.97–3.81)	1.65 (0.76–3.57)
Low ASMI †	2.05 (1.24–3.37) **	1.78 (1.04–3.02) *
High fat mass †	2.09 (1.29–3.39) **	1.92 (1.12–3.31) *

Abbreviations: OR, odds ratio; CI, confidence interval. The fully adjusted model was adjusted for age, hypertension, dyslipidemia, heart disease, osteoarthritis, osteoporosis, chronic obstructive pulmonary disease (COPD), cerebrovascular accident, diabetes mellitus, alcohol history, smoking history, Mini Nutritional Assessment-Short Form (MNA-SF) score, physical activity, and Mini-Mental Status Examination-Korean version (MMSE-KC) score. † Low hand grip strength (HGS), <28 kg; low short physical performance battery (SPPB) score ≤ 9; low appendicular skeletal muscle mass index (ASMI), <7.0 kg/m^2^; high fat mass >28.3%. * *p* < 0.05, ** *p* < 0.01.

## Data Availability

All cohort data supporting the findings of this study are available from the KFACS to all researchers upon reasonable request. All news articles published in the KFACS database, data provision manuals, and contact information were available on the KFACS website (http://www.kfacs.kr).
